# Frailty, With or Without Cognitive Impairment, Is a Strong Predictor of Future Recurrent Falls

**DOI:** 10.1093/geroni/igaa057.567

**Published:** 2020-12-16

**Authors:** Meiling Ge, Qian-Li Xue, Eleanor Simonsick, Birong Dong, Judith Kasper

**Affiliations:** 1 West China Hospital Sichuan University, Chengdu, Sichuan, China; 2 Johns Hopkins School of Medicine, Baltimore, Maryland, United States; 3 National Institute on Aging, Bethesda, Maryland, United States; 4 West China Hospital, Sichuan University, Chengdu, Sichuan, China; 5 Johns Hopkins Bloomberg School of Public Health, Baltimore, Maryland, United States

## Abstract

The associations between physical frailty and cognitive impairment with falls history are well-established. However, their associations with prospectively ascertained recurrent falls are unknown. We used data from the National Health Aging Trends Study (NHATS) and marginal means/rates model to analyze the associations between frailty and cognitive impairment and recurrent falls over 6 years (2012-2017). Of the 6,000 older adults, 1,787 (29.8%) had cognitive impairment only, 334 (5.6%) had frailty only, 615 (10.3%) had both, and 3,264 (54.4%) had neither. At baseline, compared to the group without cognitive impairment or frailty, individuals with both frailty and cognitive impairment were more likely to have a history of falls (odds ratios (OR)=2.48, 95% confidence interval (CI)=1.98-3.11), followed by those with frailty only (OR=1.53, 95% CI=1.19-1.97), and those with cognitive impairment only (OR=1.22, 95% CI=1.05-1.41), after adjusting for age, sex, race, education, living alone, obesity, comorbidity, and mobility disability. In longitudinal analysis, those with frailty and cognitive impairment alone or together at baseline had higher rates of recurrent falls than those without cognitive impairment or frailty (cognitive impairment only: rate ratios (RR)=1.07, 95% CI=1.00-1.13; frailty only: RR=1.31, 95% CI=1.18-1.44; both: RR=1.28, 95% CI=1.17-1.40). The risk appeared to be comparable between those with frailty alone or those with frailty and cognitive impairment. These findings offered robust evidence for a strong predictive association between frailty and recurrent falls in non-institutionalized older adults, and the association remained strong with or without comorbid cognitive impairment.

